# Habitual tool use innovated by free-living New Zealand kea

**DOI:** 10.1038/s41598-018-32363-9

**Published:** 2018-09-17

**Authors:** Matthew Goodman, Thomas Hayward, Gavin R. Hunt

**Affiliations:** 1 Unaffiliated, Christchurch, New Zealand; 2 Unaffiliated, Dunedin, New Zealand; 3 Unaffiliated, Auckland, New Zealand

## Abstract

The emergence of flexible tool use is rare in the animal kingdom and thought to be largely constrained by either cognitive ability or ecological factors. That mostly birds with a high level of intelligence innovate tool use in captivity is consistent with the former hypothesis. We report here the first documented case of habitual tool use innovated in the wild by a bird species only known to have used tools in captivity. Trap-boxes containing food-bait and snap-trap(s) were installed in the remote Murchison Mountains, New Zealand, from 2002 to catch introduced stoats. Kea tampered with the trap-boxes in various ways. Over approximately 2.5 years, sticks were found inserted into at least 227 different trap-boxes that were up to 27 km apart. Video footage confirmed that the stick insertion was kea tool use. Trap-boxes are unlikely to have provided the only possibility for kea tool use in their habitat given their extractive foraging and skilled object manipulation. We argue that they instead greatly facilitated the opportunity for tool use, thus increasing the chance that kea would invent the behaviour. The innovation of tool use by kea in response to facilitation provides rare field support for the cognitive constraints hypothesis.

## Introduction

The question of why flexible tool use is rare in the animal kingdom is important given the evolutionary significance of the behaviour in human evolution. The emergence, or invention, of tool use in the wild depends on both ecological and cognitive factors. Two, not necessarily mutually exclusive, ecological drivers are necessity and opportunity^1,2^. When applied to foraging, ‘necessity’, when energetic costs are taken into account^3^, promotes tool use when food obtained without tools is in short supply, and ‘opportunity’ is provided by food that can only be obtained with tools, or is more easily obtained with tools. Once tool use is initiated, it is suggested to come under genetic selection only if it is ‘profitable’, that is, when extractive foraging is on average more energetically profitable when using tools compared to not using tools^4^. All three factors may be involved in initiating and maintaining tool use in some chimpanzee *Pan troglodytes* populations^3^, but necessity may not be as important as opportunity and profitability across tool-using primate populations^2,5^.

Hypotheses for testing the association between cognition and the innovation of tool use have received less attention. Hunt *et al*.^6^ argued that there is ample ecological opportunity for tool use to evolve and proposed that the crucial constraint for its rarity across the animal kingdom is a cognitive one. The authors suggested that the innovation of tool use depends on a ‘cognitive leap’, that is, a mental comprehension that an object can be used as a tool to achieve an outcome. They also proposed that this mental understanding is more likely to come about through rapid learning while interacting with the problem at hand rather than through sophisticated cognition such as insight. The innovation of tool use will necessarily be associated with one or more underlying ecological factors that provide the potential for the behaviour^7^. Along with the mental comprehension of the utility of a tool, an individual also must manipulate the tool effectively to obtain a successful outcome, then repeat the process to maintain the behaviour.

A prediction of the cognitive-constraints hypothesis is that tool use should more likely emerge in species that have a high level of physical cognition and object manipulation skills, especially when the opportunity for the behaviour is facilitated compared to natural conditions. Captive conditions can provide such facilitation given that non-tool-using species have developed tool use in captivity^8,9^. Examples where the potential opportunity for tool use is facilitated compared to opportunities in the wild would be nutrients or food in plain view that can only be obtained with a tool or personal hygiene issues exacerbated by captive conditions that only a tool can effectively alleviate^9–11^. Captive conditions may also aid the innovation of novel behaviour through (i) food provisioning and the absence of predation, which allows increased free time and energy, and (ii) modelling, intended or unintended, of human actions^12^. Of the 21 bird species that we found in the literature reported to have used tools only in captivity, over 70% (*n* = 15) were either psittacines (parrots) or corvids (crows and their close relatives) (Table 1). The prominence of these two avian groups (ca. 5% of avian species) with their impressive cognitive and object manipulation skills^13–15^ is consistent with the above prediction. Under the ecological constraints hypothesis, species innovating tool use in captivity should represent a wide range of cognitive capabilities. However, there may be a strong bias towards psittacines and corvids innovating tool use in captivity compared to other avian species. As psittacines are commonly kept as pets and often interact with people (e.g. vocally), they are likely to be observed relatively more than other bird species. They are also probably more likely to be provided with objects such as sticks and containers for enrichment. Furthermore, researchers investigating avian cognitive abilities target psittacines and corvids for field and laboratory study. To our knowledge, there are no reports of non-tool-using birds, or other nonhuman animals, innovating habitual tool use in the wild in response to facilitation.

**Table 1:** Bird species that have used tools in captivity but were not known to be tool users in the wild.

Common name	Scientific name	Reference No.	Psittacine/corvid?
**Wedge-tailed eagle**	*Aqulia audax*	8	no
**Northwestern crow**	*Corvus caurinus*	8	yes
**Eclectus parrot**	*Eclectus roratus*	8	yes
**Yellow-crowned parakeet**	*Cyanoramphus auriceps*	8	yes
**Eurasian oyster catcher**	*Haematopus ostralegus*	8	no
**Blue-fronted Amazon parrot**	*Amazona aestiva*	8	yes
**Yellow-crowned parrot**	*Amazona ochrocephala*	9	yes
**Moluccan cockatoo**	*Cacatua moluccensis*	9	yes
**Bare-eyed cockatoo**	*Cacatua sanguinea*	9	yes
**Lesser sulphur-crested cockatoo**	*Cacatua sulphurea*	9	yes
**Sandhill crane**	*Grus canadensis*	9	no
**Bald eagle**	*Haliaeetus leucocephalus*	9	no
**Kea**	*Nestor notabilis*	9	yes
**African grey parrot**	*Psittacus erithacus*	9	yes
**Striped owl**	*Rhinoptynx clamator*	9	no
**Rook**	*Corvus frugilegus*	9	yes
**Blue jay**	*Cyanocitta cristata*	9	yes
**Marsh tit**	*Parus palustris*	9	no
**Goffin’s cockatoo**	*Cacatua goffiniana*	10	yes
**Greater vasa parrot**	*Coracopsis vasa*	11	yes
**Eurasian jay**	*Garrulus glandarius*	54	yes

Species are included if the behaviour meets two recent definitions of tool use^8,13^. However, we exclude ‘anting’ behaviour as it is relatively common in certain passerine groups and species observed anting only in captivity probably also practise the behaviour in the wild. We also exclude the endangered Hawaiian crow *Corvus hawaiiensis* because evidence suggests it used tools in the wild for extractive foraging^55^. Captive-bred Hawaiian crows were recently released into the wild at Pu’u Maka’ala Natural Area Reserve on the Island of Hawai’i, which may help to confirm whether the species uses tools under present day ecological conditions. The kea is included because the tool use that we document in the wild was innovated after individuals used tools in captivity. The table is unlikely to provide an exhaustive list of species, but clearly demonstrates the relatively high frequency of psittacines and corvids among the non-tool-using species reported to have used tools in captivity.

Parrots and cockatoos (Psittaciformes) are large-brained birds, and a sister group of passerines also with relatively large brains^16^. They have highly developed exploratory behaviour and their bill anatomy enables dexterous object manipulation^13,14,17^. Unlike most birds, psittacines can move their lower and upper bills independently from each other. However, there are surprisingly few reports of tool use by free-living psittacines. Notable cases are the use of foot-held sticks in drumming behaviour by black palm cockatoos (*Probosciger aterrimus*)^18^ and the use of wedges by several macaw species to secure hard nuts or fruit stones in the bill while they are broken (e.g. hyacinth macaws, *Anodorhynchus hyacinthinus*)^19^. Tool use in captivity demonstrates that at least some psittacines have the cognitive ability to innovate both the making and the use of tools^10,11,20^.

The kea (*Nestor notabilis*) is endemic to the South Island of New Zealand and the world’s only alpine parrot^21^. Kea are skilful at object manipulation, both in captivity and the wild^21,22^. Juvenile kea have a strong predisposition to combine objects in explorative play^22^, behaviour thought to be crucial for tool-use acquisition in children and birds^23,24^. They have used compact tools (e.g. wooden balls) and innovated stick tool use in captivity^21,25,26^, but were not known to use tools in the wild. Kea propensity for explorative manipulation of objects may be related to the challenges of finding food in a harsh alpine environment^21^. Kea are extreme foraging generalists and mainly eat fruit/seeds, nectar, leaves/leaf buds and invertebrates^21,27–29^. They practice extractive foraging in soil and vegetation using their bill^21^. In one study, over half the invertebrates kea fed on they extracted from dead wood using their strong, curved bills^28^. Therefore, it seems valid to suggest that in certain foraging situations tool use by kea might be more effective than bill use for prey extraction. Even if fine tool manipulation with a strongly curved upper bill may be challenging for kea, they are able to direct the working end of a stick tool in a functional way^30^.Thus kea should be a species with a high chance of innovating tool use in the wild.

**Figure 1:**
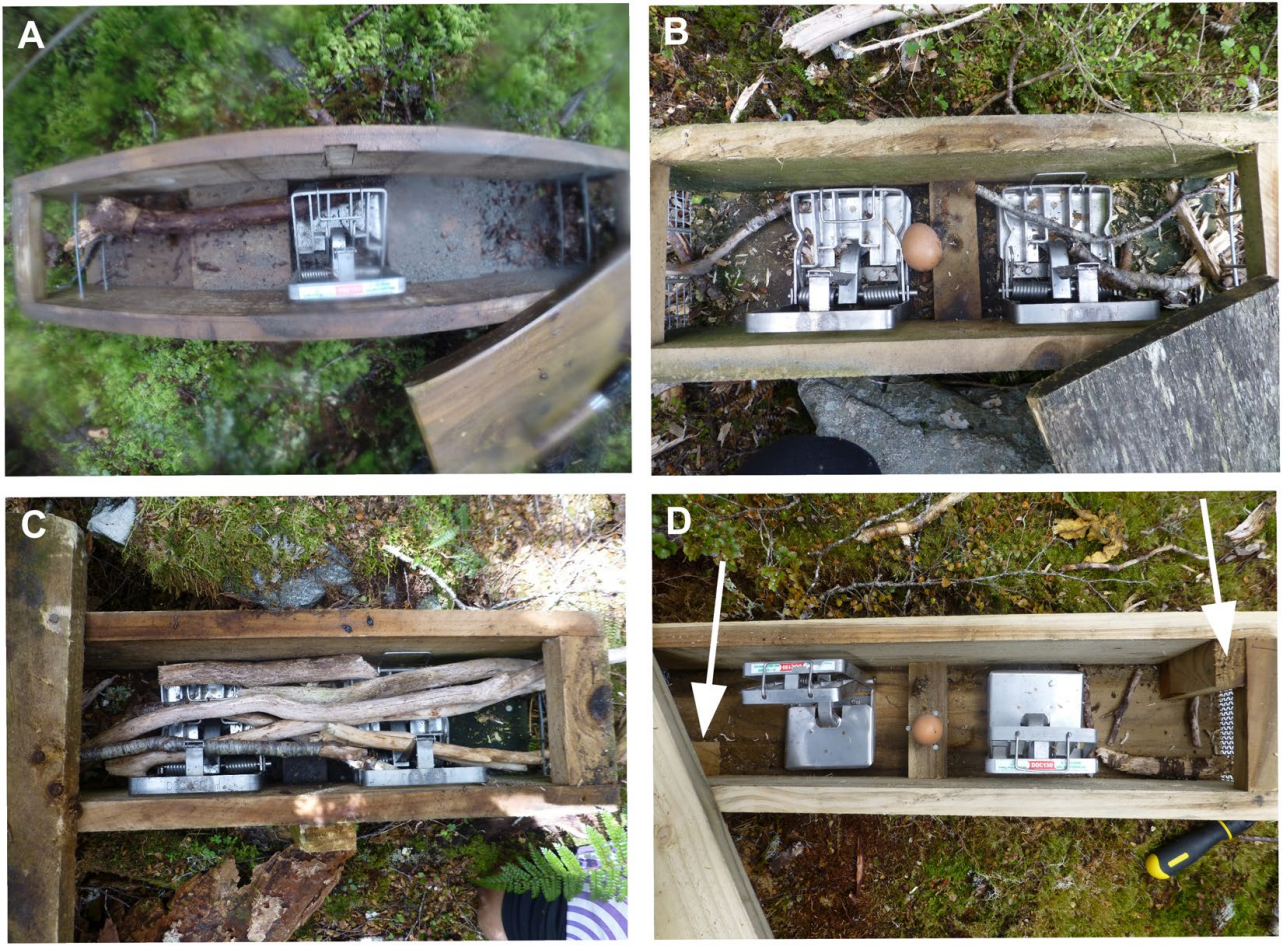
Examples of stoat traps described in the paper. (**A**) A single-set, ‘run-through’ trap-box with one DOC 150 snap-trap located between an entrance at each end of the trap-box housing. The trap-box is unbaited and has two strands of wire at each end to prevent large birds entering. A large stick has deactivated the snap-trap. (**B**) A double-set trap-box where bait is placed between two snap-traps. Sticks have been inserted into the trap-box and deactivated both snap-traps. The egg bait is unbroken. An entrance is at each end of the trap-box through different sized mesh. (**C**) An example of high stick numbers in a double-set trap-box with end entrances. Both snap-traps have been deactivated. (**D**) A double-set trap-box with side entrances like those on trap-boxes LC01 and LC02 where the kea was filmed. Meat and egg bait are positioned between the two set snap-traps. The side entrances are indicated by white arrows. Sticks have been inserted into the side entrance at right – the wooden baffle just inside the entrance appears to have prevented deactivation of the closest snap-trap. The lids on all the trap-boxes have been opened to allow inspection of the contents.

**Figure 2:**
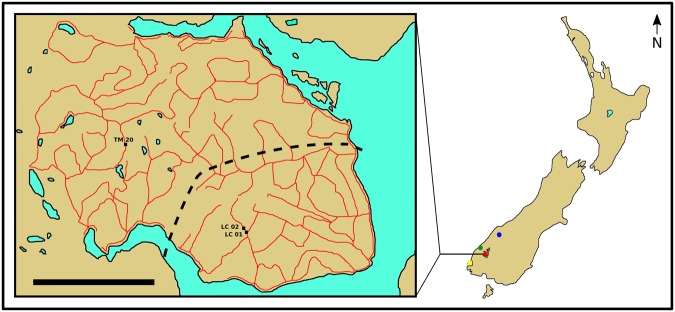
Map of the Murchison Mountains study area. The study area is located in the Fiordland National Park in the south west corner of the South Island of New Zealand (see inset). Lake Te Anau is on the eastern side of the study area. The red lines indicate the positions of trap lines where trap-boxes are currently installed ca. 100 m apart to catch stoats. The locations of the three trap-boxes where kea were filmed (TM20, LC01 & LC02) are shown on the inset map. Coloured circles positioned on the map of New Zealand show the study area (red circle) and other locations given in the text: Resolution Island (yellow), Arthur River Valley (green) and West Matukituki Valley (blue). The scale bar at bottom left on the inset is 10 km.

The Department of Conservation (DOC) has carried out intensive stoat (*Mustela erminea*) trapping operations in the south-west corner of the South Island since the early 2000s^31,32^. This successful trapping operation involved installing stoat traps on the ground at high density along trap lines in several regions. The basic early trap-box design consisted of a wooden housing with an entrance at either end in mesh or between wire strands to let stoats in but keep large birds like kea out. Snap-traps and bait (eggs and/or meat) were inside the protective housing (Fig. 1). Tampering with the trap-boxes by kea became a problem. For example, they would tip over trap-boxes that were both secured to the ground with steel pegs and unsecured^33,34^. DOC staff in the remote Murchison Mountains Special Area also started finding sticks poked into the ends of the trap-boxes that had set off snap-traps inside^34–36^ (T.H., personal observation). Stoat trapping was undertaken here from 2002 to protect the endangered ground-nesting takahe (*Porphyrio hochstetteri*)^32,33^ (Fig. 2). Many kea also live in this protected region in sub-alpine and alpine habitat. Kea were suspected of inserting the sticks into trap-boxes to try and access the bait, but there was initially no direct evidence to suggest that this was the case.

A kea habitually using sticks to probe into stoat traps in the Murchison Mountains was eventually captured on film by one of us (M.G.) using motion-activated trail cameras^37^. As far as we know, this is the only direct evidence to date that kea are responsible for inserting sticks into trap-boxes. Here we detail the kea’s probing behaviour and show that it is clearly tool use. Furthermore, the trapping operation provided an unplanned field experiment that allowed us to document the development of tool behaviour in a free-living kea population. Our findings are the first scientific documentation of a nonhuman animal using tools in New Zealand. They also have implications for explaining why flexible tool use in the wild rarely emerges in the animal kingdom.

## Results

### Development and extent of trap-box tampering by kea

**Figure 3:**
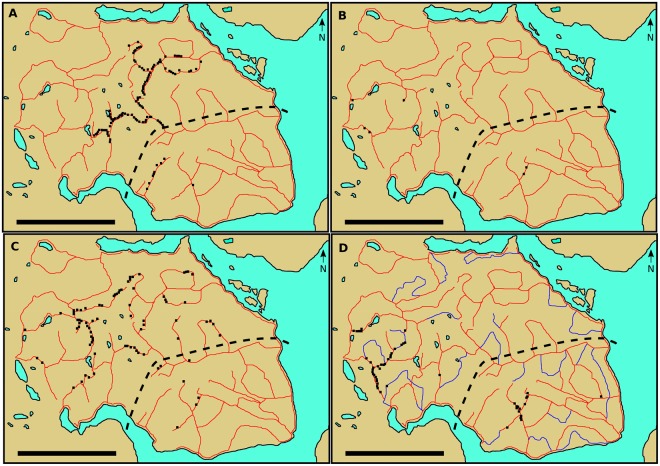
The locations of trap-boxes where sticks were inserted by kea. The black squares show the location of the trap-boxes on trap lines (red in A-C, red and blue in D) where stick insertion was recorded: (**A**) November 2010 inspection data (*n* = 113 trap-boxes), (**B**) 2011 data (*n* = 6), (**C**) January-June 2012 data (*n* = 81), and (**D**) 2014 data (*n* = 50). The region southeast of the black dashed line is where the initial 720 trap-boxes were installed from 2002 to 2007. The blue lines in D indicate the new trap lines that were installed after 2012. The scale bar at bottom left on each map is 10 km.

The 720 trap-boxes installed in the Murchison Mountains in 2002 were only systematically staked to the ground after 2007. Field workers inspecting the 720 trap-boxes before 2008 routinely found them tipped over and kea were thought to be responsible for much of this disturbance^33^. However, there is no recollection of sticks being inserted into trap-boxes before 2008 (Danilo Hegg, personal communication). Insertion of objects into the entrances of trap-boxes was noticed from 2008. The objects were not sticks but human-associated items left close to trap-boxes (batteries, wrappers and plastic tags) and stones^28^ (T.H., personal observation). Inspection staff suggested that in some cases the stones had been transported some distance to the trap-boxes as they could find none locally^36^. One of us (T.H.) first noticed the insertion of sticks into trap-boxes at the study site in 2010 (T.H. has been inspecting trap-boxes there for DOC from 2008 to the present day). A DOC report for the Takahe Programme for the period 1 July 2009 to 30 June 2010 notes that stick insertion was a significant problem, with up to 70% of snap-traps sprung in some areas^36^. Thus stick insertion by kea probably began in late 2009 or early 2010. Trap-box inspection records between November 2010 and June 2012, and in 2014, recorded sticks inserted into 113 different trap-boxes in late 2010, six in 2011, 81 in the first half of 2012, and 50 in 2014 (Table 2; Fig. 3A–D). The total of 250 stick insertion records across just over 2.5 years thus represent 227 different trap-boxes. The 227 trap-boxes were spread throughout the study area; the greatest distance between any two was ca. 27 km, and they were particularly widely dispersed in 2012 (Fig. 3C). Unbaited run-through trap-boxes were targeted for stick insertion in 2014 soon after their installation (Fig. 3D); 27 of the 50 different trap-boxes recorded with sticks inserted were this type. Stick insertion was effective at setting off snap-traps in trap-boxes as the proportion that were sprung when trap-boxes were inspected was generally much greater in those with sticks inserted (Table 2).

**Table 2:** Summary data from inspection checks on trap-boxes carried out between November 2010 and December 2014.

		Sticks inserted				No sticks inserted		
		No. of individual trap-boxes in inspection period	No. of trap-box checks/No. of snap-traps sprung and no predator capture (single snap-trap)	No. of trap-box checks/No. of snap-traps sprung and no predator capture (double snap-trap)	Total No. of snap-traps checked/% sprung	No. of trap-box checks/No. of snap-traps sprung and no predator capture (single snap-trap)	No. of trap-box checks/No. of snap-traps sprung and no predator capture (double snap-trap)	Total No. of snap-traps checked/% sprung
2010 (Nov)	250	113	24/23	89/90	202/55.9%	26/7	111/21	248/11.3%
2011 (Jan–Jun)	3924	6	none	14/0	28/0.0%	580/103	3330/591	7240/9.6%
2011 (Jul–Dec)	4049	6	none	12/2	24/8.3%	408/109	3629/1056	7666/15.2%
2012 (Jan–Jun)	6132	82	59/28	30/27	119/46%	822/183	5221/1527	11264/15.2%
2012 (Jul–Dec) and 2013	no data	no data	no data	no data	no data	no data	no data	no data
2014 (Jan–Jun)	6141	17	5/4	12/17	29/72.4%	963/15	5161/173	11285/1.7%
2014 (Jul–Dec)	5366	43	31/22	17/17	65/60.0%	556/44	4762/466	10080/5.6%
Totals	25862	n/a	119/77	174/153	467/49.3%	3355/461	22214/3834	47783/9.0%

Note that not all cases of stick insertion were recorded between November 2010 and June 2012, and in 2014.

In an effort to prevent stick insertion, smaller-sized stainless steel mesh was used to replace larger-sized galvanised mesh on the ends of some single-set trap-boxes. However, kea still succeeded in setting off the snap-traps and accessing the egg bait. They did this by removing one or two strands of stainless steel from the edge of the cut mesh and inserted it through the mesh to push the egg into the snap-trap. When the snap-trap was set off it broke the egg and kea were able to feed on the egg contents that leaked from the trap-box. This use of stainless steel strands was not observed directly, but the strands were found inserted into multiple trap-boxes (Martin Genet, personal communication; T.H., personal observation). As a consequence, the stainless steel mesh was replaced with stainless steel plate. In the areas in 2010–2011 where trap-boxes were only baited with meat to try and reduce kea tampering, kea interference was virtually eliminated, but remained high in areas where egg bait was used^38^.

Kea have also been innovative at tampering with trap-boxes without using sticks or wire. After trap-boxes were secured to the ground with steel or wooden pegs, trap-boxes were still tipped over. Kea did this by first digging around the securing pegs until they were loose enough in the ground to allow the trap-box to be tipped over. The birds also removed the lids of trap-boxes by initially removing screws securing the lids that had become loose over time. This allowed them to access the trap-box contents, which sometimes resulted in kea deaths^39^. After loose screws were secured kea then excavated wood around screws to remove lids. Stick insertion into trap-boxes and other tampering was still occurring in 2017 (M.G., personal observation). However, the latest trap-box design (i.e. with side entrances, stainless steel plate on each end, internal galvanised mesh or wooden baffles and steel brackets to protect longer screws securing the lid) has almost eliminated the deactivation of snap-traps by kea (T.H., personal observation).

Kea have tampered with trap-boxes outside the Murchison Mountains to a lesser degree. For example, they have used their bills to neatly cut holes in the fine mesh covering the ends of trap-boxes on Resolution Island, ca. 90 km away and just off the coast of Fiordland (Fig. 2), after their installation in 2008. This allowed them to get the egg bait inside (Peter McMurtrie & Sanjay Thakur, personal communications). Although there was both egg and meat bait side by side, the kea usually only took the egg bait. However, stick insertion has only been noticed in one trap-box on the island in over 10 years even though all trap-boxes there had end entrances (Peter McMurtrie, personal communication). There are unconfirmed report of sticks inserted into trap-boxes installed on the mainland relatively close to the Murchison Mountains, ca. 120 km away in the West Matukituki Valley in the summer of 2011/2012^35^. Kea have tampered with trap-boxes in other areas in Fiordland. For example, 65 km northwest of the Murchison Mountains in the Arthur River Valley kea began tipping over unsecured trap-boxes in 2005, two years after they were first installed there in 2003 (Gerard Hill, personal communication). The behaviour was rare at first then rapidly increased in frequency before the trap-boxes were secured to the ground.

### Trail camera footage of kea behaviour at trap-boxes

**Figure 4:**
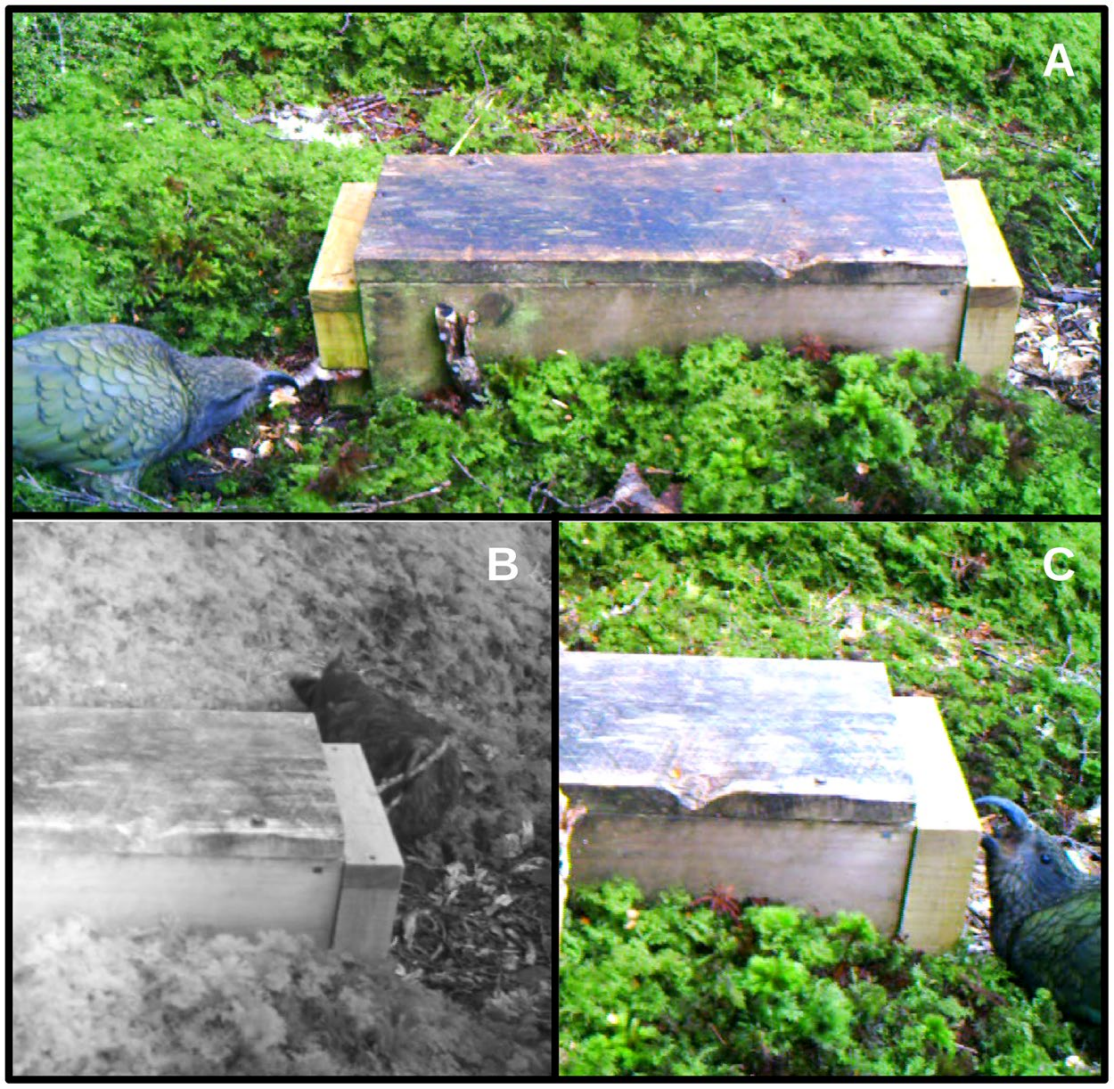
Frames from the Supplementary video footage showing the kea tampering with trap-box LC02. (**A**) The kea probing with a stick in the visible side entrance (Video S1). The lighter-coloured wood on the ends of the trap-box are modifications to move the entrances from the ends to the sides of the unit to try and prevent kea setting off snap-traps inside. (**B**) The kea probing with a stick in the side entrance on the opposite side of the trap-box (Video S4). The footage was taken in infrared because of low light conditions. (**C**) The kea biting trap-box LC02 (Video S3).

The trail cameras captured a total of 39 kea sessions at the three trap-boxes (Table 3). Except for one of the 17 sessions at TM20 when two kea were present, only one (unmarked) kea was present in a session. We assume here that all single-kea sessions at TM20 involved the same individual, and that only one individual visited adjacent LC01 and LC02. Once arriving at a trap-box, kea almost always spent their time interacting with it in a way that indicated they were interested in its contents (i.e. food if present and one or two snap-traps). This interaction occurred in three main ways. First, the kea at LC02 spent 69.9% of the time close to a side entrance involved in behaviour associated with probing into the entrance with sticks or putting its head into the entrance (observed 4 times at LC01 and 6 times at LC02). At LC01, the kea spent a small amount of time (10%) at a side entrance involved in probing behaviour as it only made one probing attempt. The kea was involved in probing behaviour in 12 of the 14 sessions at LC02. In 11 of these 12 sessions the probing was at the side entrance on the opposite side of the trap-box to where the camera was positioned (Fig. 4B, Supplementary Video S1). This meant it was not possible to directly observe all details of how sticks were manipulated in relation to that entrance. In the remaining session, the kea probed at the side entrance in direct view of the camera (Fig. 4A, Supplementary Videos S2 & S3). This confirmed that it deliberately inserted sticks into the side entrance. At LC01 (*n* = 1) and LC02 (*n* = 66), we recorded 67 cases of kea inserting, or attempting to insert, a stick into a side entrance. Interestingly, we never observed the kea attempting to insert a stick through the mesh barrier on the ends of LC01 and LC02.

**Table 3:** Details of kea behaviour at three trap-boxes obtained from trail camera footage.

Trap-box	Length of video capture period (days)/No. of actual days kea filmed	No. of sessions at a trap-box by kea	Mean session duration (min ± s.d.)	Time kea were close to trap-box (min)	Time kea were at either end of trap-box (min)	Time kea spent manipulating sticks at side entry (min)	No. of stick insertions/attempted insertions into trap-box entry
TM20	45/10	17	5.5 ± 7.2	26.7	16.8	n/a	0
LC01	28/8	8	3.4 ± 2.7	5.0	0.5	0.5	1
LC02	51/13	14	12.1 ± 12.0	76.3	3.2	53.3	66

The entries to TM20 were in the mesh on the ends of the trap-box, but the entries to LC01 and LC02 were on the sides of the trap-boxes. 47 clips with kea activity were taken at TM20, 14 clips at LC01 and 99 at LC02. All clips at LC01 and LC02 were 60 s long, but 21 of the 47 clips at TM20 were shorter than 60 s because of recording problems.

Second, kea at TM20 spent 62.9% of the time close to either end of the trap-box where they could potentially see the contents through the mesh (Table 3). They either stood in front of the mesh or looked into the mesh while standing on the lid of the trap-box. The entrances into TM20, unlike the side entrances on LC01 and LC02, were holes in the bottom of the mesh. No kea put their head into the entrance or tried to insert a stick into it. The kea visiting LC01 and LC02 spent a relatively limited amount of time in front of the ends of the trap-boxes (10% & 4.2%, respectively) (Table 3).

Last, at some stage in 28 of the 39 sessions at all three trap-boxes a kea made an apparent attempt to break into the housing. This involved using the bill to usually grasp, or bite, an edge of the wooden housing and apply some force in a levering action (Fig. 4C, Supplementary Video S4). This grasping sometimes occurred after looking into the trap-box or probing into it with a stick.

The period between the first and last recording of the kea using sticks to probe into LC02 was 51 days (probing happened on 10 of those 51 days; Table 3). The kea mostly obtained sticks within ca. 0.5 m of the side entrance in which they were used as probes. Sometimes it moved out of view of the camera and returned within a few seconds with a new stick. There was no footage of the kea carrying a stick when arriving at a trap-box. The kea was often very active when probing, moving the non-working end around a lot or removing and re-inserting the stick. The bird showed considerable persistence, with the longest probing session lasting 45 min. The persistent probing is reflected in the longer mean session times at LC02 compared to those times at TM20 and LC01 (Table 3). However, we never saw a kea obtain any food from LC02. There was only one observation of the kea setting off a snap-trap in LC02 while probing with a stick (Supplementary Video S1). When the snap-trap went off the kea immediately withdrew its stick and looked into the mesh end of the trap-box, presumably to inspect the contents. It continued probing with sticks for about eight minutes after the snap-trap was deactivated. The kea once left the side entrance it was probing in, went quickly over the trap-box to put its head in the other side entrance, then quickly back again to probe in the original entrance. This rapid movement between side entrances also indicated that the kea was focused on the trap-box’s contents.

There were four cases of the kea throwing away the stick it was probing with and then selecting another stick to use. The kea also sometimes withdrew the stick it was probing with, flipped it around then used it to probe again, but it was unclear if the flipping was intentional. The kea also used its bill to modify probing sticks, either by shortening/removing side extensions (in 3 sessions) (Supplementary Video S2) or removing material from larger diameter sticks (in 4 sessions) (Supplementary Video S5).

Kea play extensively with objects^21^. In the last five sessions at LC02, the kea appeared to play with sticks in an obvious way. These five sessions occurred over the 17 days at the end of the 51 day period when the kea was filmed probing with sticks. The play was distinctly different to the kea’s use of sticks to probe into the trap-box as it occurred away from a side entrance. The play sometimes involved the kea throwing a stick with its bill then picking it up again, carrying it away from the trap-box or manipulating a stick while lying on the ground (Supplementary Videos S4 & S6). The kea also threw sticks away after probing with them, but these cases appeared to be related to frustration from a lack of probing success. The kea at LC01 also appeared to play with a triangular white-plastic marking tag that it had found, on three occasions carrying it from the ground to the top of the trap-box.

## Discussion

The main reason why kea tampered with baited trap-boxes was to access the egg bait, both inside and outside our study area. Our video footage demonstrated that the repeated stick insertion and probing by the kea at LC02 was purposeful to interact with out-of-reach trap-box contents. Also, the kea at TM20 was intent on looking into the trap-box. Kea are known to persist at exploratory play with an object^21^, but the obvious play at LC02 did not involve stick insertion, or probing, into the side entrances. Thus the kea’s stick probing at LC02 was purposeful and clearly tool use^8,40^. The tool use has important implications for the unobserved insertion of objects (i.e. sticks and wire) into trap-boxes both inside and outside the Murchison Mountains study area. Kea also probed into unbaited run-through traps-boxes in 2014 (Fig. 1A). This is curious given that they seemed to have previously ignored trap-boxes without egg bait several years earlier. Thus kea might have probed into run-through trap-boxes that they probably knew were unbaited because the effect of setting off snap-traps was intrinsically rewarding. They may have done so as the snap-traps were relatively easy to reach with sticks through entrances on the ends of these trap-boxes. Further work is needed to determine if kea used tools to simply deactivate snap-traps.

Kea stick tool use described here appears to have been innovated in response to the introduction of trap-boxes. First, tool use by free-living kea was previously unknown in spite of considerable research on their behaviour in the wild^21^. This is supported by the lack of stick insertion into trap-boxes over many years on Resolution Island and at other sites in and close to Fiordland. Second, the first sticks inserted into trap-boxes were only noticed in the study area well after the initial introduction of (baited) trap-boxes in 2002. If the kea were already proficient at using stick tools, we would have expected to find sticks commonly inserted into trap-boxes soon after their initial installation in 2002 given the kea’s extractive foraging skills and curiosity about human-associated objects. However, as Fig. 3 shows, sticks were recorded inserted into relatively few trap-boxes in the south east region where traps were restricted to from 2002–2007. The original trap-boxes in this region were not modified with side entrances until after 2012 (Supplementary Table S1). Last, the first items noticed inserted into trap-boxes were not sticks but those that were ineffective at setting off snap-traps or aiding bait extraction. Given anecdotal reports of sick insertion into trap-boxes 120 km distant from the study area, tool use by kea to access trap-box contents may have developed independently more than once.

Large variation across years existed in the number of different trap-boxes in the study area that were recorded with sticks inserted. For example, the low number in 2011 contrasts with the much higher numbers in November 2010 and January-June 2012. Some of the variation was due to missing data when staff inspecting trap-boxes did not record stick insertion. Variation may also be associated with the series of modifications to the design of trap-boxes to try and prevent kea tampering (Supplementary Table S1). The initial outbreak of stick insertion in 2009/2010 was quickly followed by the fitting of wooden baffles to the side entrances of trap-boxes outside the south-east region. The baffles likely reduced stick insertion in 2011 before it seems to have increased in January-June 2012. Modifications to trap-boxes such as fitting side entrances also reduced the frequency of kea stick insertion, especially just after their introduction (T.H., personal observation). The widespread tampering with trap-boxes at several Fiordland locations that did not involve sticks (e.g. tipping over trap-boxes, opening trap-box lids) was carried out by many kea. Similarly, the widespread stick insertion across our study area suggests that more than one kea were inserting sticks into trap-boxes. Data on kea movements show that individual kea could potentially forage throughout much of the Murchison Mountains. Daily activity patterns of two banded kea at Mt Cook National Park included flights of 10–20 km^29^. Studies^41,42^ in the central and upper South Island also found that banded kea were usually resighted 10–20 km from their banding location. Juveniles, though, probably disperse from their natal range when independent^42^. Nevertheless, the extent, geographic spread and persistence of stick insertion into trap-boxes over many years makes it more likely that multiple kea were habitually carrying out this behaviour.

At first glance, our findings might be viewed as support for the predominant role of ecological conditions in initiating tool use. For example, there was no ecological opportunity for kea to use probing tools before the trap-boxes were installed. The trap-boxes provided that opportunity and tool use was invented. Similarly, food supplies could have been unusually scarce around the time kea began inserting sticks into trap-boxes, thus necessity together with opportunity might have initiated tool use. Few data exist on how kea food supplies varied in the study area. However, many plants in New Zealand forests, including the extensive beech forests (family Nothofagaceae) in the Murchison Mountains^32^ (kea are known to occasionally eat beech seed^29^), are masting species and do not produce an abundance of sound seed every year^43^. Therefore, beech mast years in our study area may provide some indication of weather-related variation in a range of food resources for the birds. There were four mast years between 2000 and 2015: in 2006/2007, 2009/2010, 2011/2012 and 2014/2015 (Sanjay Thakur, personal communication). This means there were at least four non-mast years after trap-boxes were first installed in 2002 before kea began to probe into trap-boxes with sticks. Thus the initial appearance of kea tool use around 2009/2010 does not appear to be closely associated with extremely low food resources in their habitat (necessity hypothesis).

Cognitive constraints may better explain why kea did not appear to have invented tool use when foraging at our study site before the introduction of trap-boxes. Our discussion below focuses on cognitive constraints and the invention of tool use at the individual level and not on evolutionary explanations. First, Kea practice extractive foraging and are highly motivated to interact with potential probe sites. In captivity they are much more interested in a cube with a slot that can be probed with the bill or looked into than one without a slot^21^. In their sub-alpine forest habitat, there should be potential probe sites with cached food (e.g. invertebrates, lizards) where it would be more efficient to employ tool use. Although it has a bill specifically evolved for fine tool manipulation^44,45^, the New Caledonian crow uses tools extensively to extract prey cached in forest habitat from near sea level to over 1,600 m above sea level^46,47^. Thus it is highly likely that opportunities exist for kea to invent tool use to extract prey from natural probe sites. Furthermore, the probing by New Caledonian crows is often more investigative and does not appear to require precision manipulation of the end of the tool (G.R.H., personal observation). We have shown here that kea have the manipulation skills to carry out this kind of tool use. Bills of female kea are 13% shorter than those of males^48^, thus tool use by females in particular may be advantageous for obtaining out-of-reach prey. Second, the design of the initial trap-boxes facilitated the opportunity for innovating tool use compared to the characteristics of natural holes and crevices. The trap-boxes made it relatively easy for a bird with high object manipulation skills to get a successful outcome by inserting a stick (i.e. access to food). Trap-boxes with end entrances in wide mesh, in particular, enabled kea to easily insert sticks of a range of sizes while having a clear view of the ‘cached’ contents (i.e. food and snap-trap) and stick movement. In contrast, potential prey cached in natural probe sites are unlikely to be in full view of kea. Side entrances where kea could not see trap-box contents while probing were probably more realistic of natural probe sites. However, probing into side entrances likely occurred after previous experience of probing into the ends of trap-boxes. The trap-boxes were also at high density, visually obvious and regularly baited over many years, which provided the opportunity for kea to repeat and maintain the tool use. Third, the lack of tool use by kea to probe into trap-boxes outside the study area is inconsistent with the ecological opportunity hypothesis. Kea probing into trap-boxes with sticks across several years in the Murchison Mountains suggests that tool use was often advantageous for obtaining egg bait. Their behaviour at other locations, such as biting through mesh to extract egg bait and tipping over trap-boxes, demonstrates that the kea were well motivated to interact with trap-box contents. Thus those trap-boxes clearly provided an opportunity for kea tool use but evidence of the behaviour only consists of anecdotal reports.

To recall, natural probe sites likely exist for kea where tool use to extract prey would be more efficient than bill use, even if they were uncommon. The trap-boxes at our study site greatly facilitated tool use, but even so the behaviour did not emerge until many years after their installation. This reinforces our contention that innovating tool use at natural probe sites is cognitively demanding. Confirmation that the trap-boxes facilitated the innovation of kea tool use as opposed to providing a unique opportunity for the behaviour would be if kea transferred the tool use to natural probe sites when extractive foraging. Therefore, follow up work on natural foraging by kea in the Murchison Mountains could provide important insight into why tool use is rare in animals. That tool use emerged at our study site but has been rarely reported at other trapping locations suggested that some kea have a level of domain-general intelligence for inventing tool use while others do not, which also implicates cognitive constraints. Under the profitability hypothesis^4^, when tool use is more efficient, on average, than bill use it will evolve to become population-level behaviour. For example, a genetic basis for tool use is evident in several tool-using bird species, including the New Caledonian crow^4,44^, but there are currently few data to suggest that tool use is under selection in nonhuman mammals^49^. Our findings raise the distinct possibility that tool use could persist as occasional behaviour in certain species without coming under selection. Under this scenario, kea with the domain-general intelligence to invent or socially acquire the behaviour would use tools at those less common probe sites where bill use was ineffective. That only some kea appear to have this level of domain-general intelligence even when the opportunity for tool use is greatly facilitated, combined with the increased cognitive challenges of inventing tool use at natural probe sites, would place substantial cognitive constraints on tool use emerging in the course of natural foraging.

Field experiments with honey extraction also implicate cognitive constraints as the main reason for the lack of stick tool use by some primate populations who only use other types of tools. The Sonso population of chimpanzees in Uganda that use leaf tools but not stick tools failed to use sticks to extract honey in prepared holes^50,51^. In a similar experiment, the Pedra Furada group of bearded capuchin monkeys (*Sapajus libidinosus*) in Brazil who use stone tools but not stick tools also failed to use sticks to extract honey from holes^52^. When the degree of facilitation was increased by leaving sticks in the holes for individuals to pull out and use as tools, individuals from both populations still failed to use the sticks as tools^51,52^. Gruber and colleagues^3,50,51^ have suggested that conservatism or cultural bias for existing, non-stick-tool behaviour and/or ‘functional fixedness’ (failure to use familiar objects in novel ways) may have prevented stick tool innovation in Sonso chimpanzees. Similar work with captive, non-tool-using macques (*Macaca fascicularis fascicularis*) failed to elicit stone-hammering tool use to access nut contents in 31 motivated individuals^53^. This was in spite of increasing levels of facilitation that culminated in a full tool use demonstration by their human keeper. Further research is needed to identify the cognitive constraints that can prevent experienced tool-users inventing novel tool use. Nevertheless, the above examples lend support to the view^6^ that cognitive constraints rather than associated ecological conditions are the crucial factor limiting the innovation of flexible tool use in nonhuman animals. Our study was similar to the capuchin and chimpanzee field experiments as kea could also decide on their own whether or not to participate, but it was an unplanned one. Unlike the primate experiments above, the degree of facilitation for tool use was not increased for kea. On the contrary, it was mostly reduced in response to kea success at inserting sticks into trap-boxes and setting off snap-traps.

In summary, we provide the first evidence that a non-tool-using bird species with a high level of physical cognition innovated habitual tool use in the wild in response to facilitation. This finding is consistent with a prediction of the cognitive constraints hypothesis explaining why flexible tool use is rare. That is, birds with a high level of intelligence and physical cognition, like the kea, should more readily innovate tool use when it is facilitated. We suggest an experimental paradigm to test this prediction, where non-tool-using species that differ in cognitive ability would be required to innovate tool use that is facilitated to varying degrees. The expectation would be that the level of physical cognition and object manipulation skills will be negatively correlated with the degree of facilitation needed for tool use to emerge. Furthermore, our findings indicate that even with a high degree of facilitation the invention of tool use in the wild for species like the kea with considerable domain-general intelligence is cognitively demanding.

## Methods

### Trapping regime

The study site was the 510 km^2^ Murchison Mountains Special Area of the Fiordland National Park (Fig. 2). Access to the public is severely restricted, being limited to the McKenzie Burn Valley and Woodrow Burn in the far west of the study area and only for a short period each year. Thus only research and conservation personnel visit the majority of the study area. Trap lines were installed on the peninsular from 200 m a.s.l. on the edge of Lake Te Anau to over 1,500 m a.s.l. From 2002 to 2007, 720 trap-boxes were installed in the south-east sector of the study site over an area of ca. 150 square km^32,33^ (see Fig. 3). This number was increased to 1745 trap-boxes in the summer of 2008/2009 throughout most of the 510 km^2^ study site^32^. Between 2013 and 2015, the number of trap-boxes was increased to 3400 by adding new trap lines and infilling existing trap lines so trap-boxes were 100 m apart rather than 200 m apart^32^. The infilling took place from 300 m a.s.l. to the bush line. Twenty-two new trap lines were added and an existing one was extended (Fig. 3D). We plotted the GPS locations of trap-boxes onto a 1:25,000 contour map using the software Memory-Map Version 6.

The trapping system used from 2002 to 2007 was a double-set trap-box, consisting of bait placed between two Fenn Mark 4 snap-traps (see Fig. 1B–D where DOC 150 snap-traps are used instead of the Fenn Mark 4 snap-traps). The Fenn snap-traps were replaced with DOC 150 snap-traps in 2007/2008. Fenn snap-traps close upwards, in contrast to the DOC 150 snap-traps which close downwards. It is possible that an inserted stick was more likely to set off, and get caught in, a DOC 150 snap-trap than a Fenn Mark 4 trap (T.H., personal observation). Trap-boxes installed after 2007 were mostly double-set containing two DOC 150 snap-traps. Some baited single-set trap-boxes were installed in 2008/2009 in alpine areas above the bush line that contained a single DOC 150 snap-trap. These trap-boxes had a side entrance at only one end of the unit. That is, there was only one snap-trap and the egg bait was between the snap-trap and the end without an entrance. The original 13 mm galvanised mesh on these traps was replaced with smaller-sized stainless steel mesh to try and prevent stick insertion, before being replaced with stainless steel plate. In 2013/2014, 100 unbaited single-set, ‘run-through’ trap-boxes were also installed below the bushline (see Fig. 1A). These ‘run-through’ trap-boxes only had two horizontal wire strands at each end, allowing small mammals to easily pass through the trap-box and potentially trigger the snap-trap. Run-through trap-boxes had been successful at catching stoats at other locations. The DOC 150 snap-traps were activated when a weight similar to that of a stoat was placed on a pressure plate.

Stoats could enter most trap-boxes from both ends, but the design of the entrance varied in three main ways. Early trap-boxes and later ‘run-through’ trap-boxes had entrances at each end through which sticks could easily be inserted (Fig. 1A–C). To try and prevent stick insertion, the ends were closed off with small-sized galvanised or stainless steel mesh and a side entrance was installed at each end of the housing (Fig. 1D). The side entrance made it more difficult for an inserted stick to set off the snap-trap inside. The mesh on the 1745 trap-boxes installed in 2002 and 2008/2009 began to be replaced with stainless steel plate to prevent the insertion of objects into the ends of trap-boxes (Supplementary Table S1). The interior of all trap-boxes was clearly visible through the mesh barriers, but not when the stainless steel plate was installed. From their first installation in 2002, trap-boxes were inspected, and baited where necessary, usually quarterly in August, November, February and May^32^. We only obtained (incomplete) trap-box inspection records for the period between November 2010 and December 2014. In this approximately 4-year period the insertion of sticks was only noted on some trap-box inspection records. Stick insertion was not recorded for inspections between July 2012 and December 2013. Also, from November 2010 to June 2012, and in 2014, stick insertion was not recorded for all trap lines. Thus the stick insertion data we present here provides a minimum account of the number of trap-boxes that kea probed into and the frequency of the probing behaviour.

Trapping staff also manipulated the bait inside trap-boxes to investigate if kea were using sticks to try and access the egg bait rather than the meat bait. In the summer and autumn of 2010–2011, all trap-boxes above the bush line on around seven trap lines were only baited with meat and eggs were not used^38^.

### Collection of trail camera footage

Stick insertion into trap-boxes was common along a 2.5 km section of trap line in Mystery Burn Valley in the south-east corner of the study area. Trail cameras were installed at trap-boxes along this section and at one trap-box close to an accommodation hut 12 km away (Fig. 2). The cameras were motion-triggered and were set to record video footage in 1 min clips after detecting movement until it ceased. Five cameras were destroyed by kea and the memory cards were missing. Video footage was obtained at three trap-boxes, and in each case kea visits were filmed: TM20 near the hut above the tree line at 1160 m a.s.l. in the summer of 2013/2014, and LC01 and LC02 at 880 m a.s.l. in mountain beech forest (*Fuscospora cliffortoides*) in the summer of 2014/2015. LC01 and LC02 were 100 m apart. TM20 had one end entrance and contained only one snap-trap, and LC01 and LC02 had side entrances and contained two snap-traps (see Fig. 1D). All three trap-boxes were baited with food twice during the summer, in November/December and February.

From the video footage we recorded (1) the total time a kea was within ca. 30 cm of a trap-box, (2) the length of a session, or visit, to a trap-box by a kea. Sessions were separated by no kea presence in video footage for more than 15 min, (3) the length of time a kea spent in front of either end of a trap-box, (4) the length of time a kea spent manipulating a stick at the side entrance of LC01 and LC02, (5) whether a kea used its bill to bite the trap-box housing, (6) whether a kea inserted its head into a trap-box entrance, (7) manipulation of a stick by modifying it, carrying it more than ca. 1 m, flipping it or exchanging it for another stick, (8) the insertion of a stick into a trap-box, and (9) probable play activity.

## Electronic supplementary material

Video S1

Video S2

Video S3

Video S4

Video S5

Video S6

Supplementary material

## Data Availability

The datasets generated and/or analysed during the current study are available from the corresponding author (G.R.H.) on reasonable request, and after any necessary permission has been granted from the New Zealand Department of Conservation.
