# Supplementary material for: Habitual tool use innovated by free-living New Zealand kea
Source: Sci Rep. 2018 Sep 17;8:13935. doi: 10.1038/s41598-018-32363-9 (PMC6141560; doi:10.1038/s41598-018-32363-9)
Supplement: Supplementary file 7 — Supplementary material [file 41598_2018_32363_MOESM7_ESM.docx]

**Habitual tool use innovated by free-living New Zealand kea**

Matthew Goodman^1,*^, Thomas Hayward^2^ & Gavin R. Hunt^3,*^

^1^Unaffiliated, Christchurch, New Zealand

^2^Unaffiliated, Dunedin, New Zealand

^3^Unaffiliated, Auckland, New Zealand

***: Correspondence and requests for materials:**

Email: [grhunt10@hotmail.com](mailto:grhunt10@hotmail.com)

Email: [matgoodman18@gmail.com](mailto:matgoodman18@gmail.com)

**Supplementary Table S1**

| **Trap-box details** | **Trap-box installation dates** | | |
| --- | --- | --- | --- |
|  | **2002** | **2008/2009** | **2103-2015** |
| **Number installed** | 720 | 1025 | 1655 |
| **Location** | Southeast region in new trap lines | Outside southeast region in new trap lines | Throughout study area in new and existing trap lines |
| **Design when installed** | End entrances in mesh, no baffles to try and prevent probing sticks reaching snap-traps | Side entrances, ends covered with mesh, no baffles | (A) 1555 trap-boxes with side entrances, ends with stainless steel plate, and baffles next to snap-traps. (B) 100 ‘run-through’ trap-boxes with entrances through each end |
| **Type of snap-trap when installed** | Fenn Mark 4 (replaced with DOC 150 snap-traps in 2007/2008) | DOC 150 | DOC 150 |
| **Entrance modifications** | 2013-2014: changed to side entrances with mesh baffles at entrances | 2010-2011: wooden baffles fitted to entrances | None |
| **End modifications** | 2013-2014: mesh replaced with stainless steel plate | 2016-present: mesh replaced with stainless steel plate | None |

**Table S1. Details and design characteristics of trap-boxes installed in the Murchison Mountains Special Area.**

**Supplementary Video legends**

**Video S1.** Kea setting off a snap-trap inside trap-box LC02 (27/1/15). Most of the clip shows the kea actively probing with a large stick inserted into the side entrance on the far side of the trap-box. The probing then sets off a snap-trap inside the trap-box. This is indicated by the movement of the trap-box and the startled behaviour from the kea. The kea then immediately removes the stick and looks into the trap-box through the mesh covering the end of it. It then returns to the side entrance and possibly puts its head into the entrance.

**Video S2. Kea using a stick tool to probe into trap-box LC02 (25/2/15)**. In this clip the kea appears to deliberately modify the tool before it is inserted into the trap-box entrance. It first breaks off a side twig then removes a branched section at the narrow end of the tool. It breaks another piece off the narrow end after inserting the wide end of the tool. The clip clearly shows the kea inserting the stick into the side entrance of the trap-box.

**Video S3. Kea using a stick tool to probe into trap-box LC02 (25/2/15).** The kea inserts the narrow end of the tool into the side entrance of the trap-box. It then manipulates the tool, moving it in different angles and short distances in and out of the trap-box. From the footage, it appears that the tool is angled into the trap-box and that the working end is probably past the wooden baffle just inside the entrance.

**Video S4.** **Kea using its bill in an apparent attempt to break into trap-box LC02 (10/2/15).** The kea initially seems to play with the stick. It is importantly doing this away from the side entrance on the righthand side of the far side of the trap-box and does not attempt to insert the stick into the trap-box. Then it bites the edge of the trap-box, plays with the stick again and bites the trap-box edges again before leaving the frame. When biting while standing on the ground the trap-box moves, indicating that the kea is exerting considerable force on the trap-box. The kea’s behaviour suggests that it wants to get into the trap-box.

**Video S5.** **Kea modifying a stick at trap-box LC02 (9/2/15).** The kea uses its bill to bite material off one end of a stick that it has been probing with in a previous clip.

**Video S6.** **Kea playing with a stick at trap-box LC02 (10/2/15)**. The kea makes a brief and ineffectual attempt to insert a thin stick into the visible side entrance. It then plays with a short, wide section of a branch. As in Video S3, it is doing this away from a side entrance and makes no attempt to insert the stick into the trap-box.
